# Ascertaining asthma status in epidemiologic studies: a comparison between administrative health data and self-report

**DOI:** 10.1186/s12874-023-02011-6

**Published:** 2023-09-07

**Authors:** Marie-Claude Rousseau, Florence Conus, Mariam El-Zein, Andrea Benedetti, Marie-Elise Parent

**Affiliations:** 1 Epidemiology and Biostatistics Unit, Centre Armand-Frappier Santé Biotechnologie, Institut national de la recherche scientifique (INRS), Laval, QC Canada; 2 School of Public Health, Université de Montréal, Montréal, QC Canada; 3 Present Address: Direction des enquêtes de santé, Direction principale des statistiques sociales et de santé, Institut de la statistique du Québec, Montréal, QC Canada; 4 Present Address: Division of Cancer Epidemiology, McGill University, Montréal, QC Canada; 5 Respiratory Epidemiology and Clinical Research Unit, Research Institute of the McGill University Health Centre, Montréal, QC Canada; 6 Department of Epidemiology, Biostatistics and Occupational Health, Faculty of Medicine, McGill University, Montréal, QC Canada

**Keywords:** Administrative health data, Agreement, Asthma, Self-report

## Abstract

**Background:**

Studies have suggested that agreement between administrative health data and self-report for asthma status ranges from fair to good, but few studies benefited from administrative health data over a long period. We aimed to (1) evaluate agreement between asthma status ascertained in administrative health data covering a period of 30 years and from self-report, and (2) identify determinants of agreement between the two sources.

**Methods:**

We used administrative health data (1983–2012) from the Quebec Birth Cohort on Immunity and Health, which included 81,496 individuals born in the province of Quebec, Canada, in 1974. Additional information, including self-reported asthma, was collected by telephone interview with 1643 participants in 2012. By design, half of them had childhood asthma based on health services utilization. Results were weighted according to the inverse of the sampling probabilities. Five algorithms were applied to administrative health data (having ≥ 2 physician claims over a 1-, 2-, 3-, 5-, or 30-year interval or ≥ 1 hospitalization), to enable comparisons with previous studies. We estimated the proportion of overall agreement and Kappa, between asthma status derived from algorithms and self-reports. We used logistic regression to identify factors associated with agreement.

**Results:**

Applying the five algorithms, the prevalence of asthma ranged from 49 to 55% among the 1643 participants. At interview (mean age = 37 years), 49% and 47% of participants respectively reported ever having asthma and asthma diagnosed by a physician. Proportions of agreement between administrative health data and self-report ranged from 88 to 91%, with Kappas ranging from 0.57 (95% CI: 0.52–0.63) to 0.67 (95% CI: 0.62–0.72); the highest values were obtained with the [≥ 2 physician claims over a 30-year interval or ≥ 1 hospitalization] algorithm. Having sought health services for allergic diseases other than asthma was related to lower agreement (Odds ratio = 0.41; 95% CI: 0.25–0.65 comparing ≥ 1 health services to none).

**Conclusions:**

These findings indicate good agreement between asthma status defined from administrative health data and self-report. Agreement was higher than previously observed, which may be due to the 30-year lookback window in administrative data. Our findings support using both administrative health data and self-report in population-based epidemiological studies.

**Supplementary Information:**

The online version contains supplementary material available at 10.1186/s12874-023-02011-6.

## Background

Asthma diagnosis is based on clinical history, physical examination, and the assessment of markers of lung function such as airway hyperresponsiveness, peak expiratory flow variability and bronchodilator reversibility [1]. Objective verification of asthma cannot realistically be applied in large population-based studies, and many alternative sources of information are used for identifying persons with asthma including clinical examination, medical chart review, self-report, and administrative health data [2–5].

Self-report and administrative health data are often the most convenient and available sources of data for ascertaining asthma status in large epidemiological studies. In Canada, most of the national estimates of asthma prevalence are derived from self-reported data, such as the National Longitudinal Survey of Children and Youth (NLSCY) [6] and the Canadian Community Health Survey (CCHS) among adults [7]. Administrative health data are increasingly used for conducting population-based epidemiological studies through linkage with demographic, clinical, and other datasets, allowing to identify asthma cases and estimate asthma prevalence and incidence [8–13]. Asthma status defined from self-report and administrative health data have been compared with medical records, and found to be valid in North America and Europe [9, 14–19].

Studies have documented the agreement between administrative health data and self-report for identifying individuals with asthma. The observed Kappas were fair to good, varying from 0.27 to 0.62 [20–28]. Agreement was slightly higher for youth (12–18 years) than adults [21]. Previous studies differed in terms of the lookback windows (the retrospective period during which administrative health data were considered) and the definitions applied. Some studies investigated the impact of varying lookback windows (1, 2, 3 or 5 years) on agreement [20–23], whereas others selected only one (not always the same) [24, 26, 28], or applied a definition that had to be met within a 2-year time interval in longer lookback windows (10–15 years) [25, 27]. However, to our knowledge, none of the previous studies has specifically compared administrative health data covering several decades with self-reported ever asthma in adulthood. This is highly relevant since neither source is considered to be a gold standard, yet both are used in epidemiological studies.

Further, few studies have identified the determinants of agreement between administrative health data and self-reported asthma status. Some of the reported determinants of higher agreement include sex and age, both with inconsistent findings, absence of comorbid conditions, as well as higher levels of income and education [21, 23, 27, 28].

In this context, we aimed to (1) evaluate agreement between administrative health data from childhood to adulthood and self-report in adulthood of ever having asthma, and (2) identify determinants of agreement between these two sources for a person’s asthma status.

## Methods

### Study design and population

We used administrative health data from 1983 to 2012 from the Quebec Birth Cohort on Immunity and Health (QBCIH) which was originally designed to examine an association between bacillus Calmette-Guerin (BCG) vaccination and childhood asthma occurrence [29]. Briefly, this population-based birth cohort was assembled through probabilistic linkage of provincial administrative databases and included 81,496 subjects born in the province of Quebec, Canada, in 1974 at or after 32 weeks of gestation. Administrative data were extracted from the birth, BCG vaccination and death registries, and the Healthcare Registration File (universal public health system). Health services were obtained from physician billing claims for consultations (starting in 1983) and hospitalization data (starting in 1987) until 2012.

In 2012, we conducted the Survey on Childhood Environment and the Development of Allergic Diseases. A detailed description of this methodology can be found elsewhere [30]. Telephone interviews were conducted with a subset of the QBCIH subjects (n = 1643) using a two-stage sampling strategy with a balanced design [31]. Subjects were randomly selected among 4 strata defined by cross-tabulating BCG vaccination (Yes/No) and childhood asthma status based on administrative health data (Yes/No), and a similar number of participants per stratum was recruited [31]. For sampling, persons were considered to have asthma if they had ≥ 2 asthma-related physician claims or ≥ 1 asthma-related hospitalization until 1994 (20 years of age). The participation rate among persons invited for the survey was 56% [31]. The analytical sample for the present project included the 1643 telephone interview participants. The QBCIH and the Survey on Childhood Environment and the Development of Allergic Diseases were approved by ethics committees at *Institut national de la recherche scientifique*, *Institut de la statistique du Québec* and *Régie de l’assurance maladie du Québec* (RAMQ), as well as the *Commission d’Accès à l’Information* of Quebec. Telephone interview participants gave a verbal informed consent.

### Asthma definition in each data source

In the administrative health databases, identification of subjects with asthma was based on diagnostic code 493 from the International Classification of Diseases (ICD)-9th revision for all physician claims and for hospitalizations until 2005, and code J45 from the ICD-10th revision for hospitalizations from 2006. Healthcare encounters were considered until the time of interview, in 2012. If there was more than one claim per day, only one claim was counted. If a hospitalization and a physician visit presenting an ICD code for asthma were both present, the hospitalization was counted. To facilitate comparisons across studies, we applied five definitions of asthma by varying the time interval in which they had to be met, over our 30-year lookback window: ≥2 asthma-related physician claims within 1, 2, 3, 5, or 30 years or ≥ 1 asthma-related hospitalization. There is no universal gold standard among these definitions, however the Canadian Chronic Disease Surveillance System defines prevalent asthma as consisting of: ≥2 asthma-related physician claims within a 2-year period or ≥ 1 asthma-related hospitalization ever [32].

Using self-report, the identification of subjects with asthma was based on the following questions: (1) “Have you ever had asthma?”, if yes; (2) “Was your asthma diagnosed by a physician?”. We created two corresponding variables, namely “ever had asthma” and “ever had asthma diagnosed by a physician”.

### Determinants of agreement for asthma status

We considered variables that were either documented in administrative databases or collected at interview. From the former source, we included sex, language (French/English), parental place of birth (both in Quebec/both outside Quebec/in and outside Quebec), area of residence in 1987, 1991 and 2011 (all urban/all rural/urban and rural; based on the 2nd character of the subjects’ postal codes) [33], family income in 1991 and 2011 (average quartile of median family income from the Canadian census, rounded up to the nearest integer; based on the first three characters of the subjects’ residential postal codes), area-based material and social deprivation indices in 1987, 1991, and 2011 (average quintile based on the subjects’ residential postal codes, rounded up to the nearest integer), and number of health services for allergic diseases other than asthma, including allergic rhinitis, eczema, allergic urticaria, and other allergies unspecified (0/≥1). Variables collected at interview included the highest level of education attained by the participants’ mother and father (elementary school/secondary school/college/university), as well as parental history of asthma (no/yes).

### Statistical analysis

We estimated the proportion of overall agreement, Kappa coefficient, proportions of positive and negative agreement, comparing each of the five asthma definitions from administrative health data with self-reported ever asthma and physician diagnosis of asthma. The sample was weighted according to the inverse of the selection probability to correct for bias introduced by the stratified sampling. We calculated the sampling probabilities using the BCG-asthma 2 × 2 table from the cohort and the equivalent 2 × 2 table from the survey participants. The sampling probabilities were the same for all subjects within each of the four strata and corresponded to n_survey_/n_cohort_. The variance estimates used to calculate the 95% confidence intervals (CIs) were based on the actual numbers of survey participants. Kappa indicates the proportion of agreement beyond that expected by chance. Levels of agreement for Kappa were considered poor when < 0.20, fair at 0.20–0.39, moderate at 0.40–0.59, good at 0.60–0.79, and very good at 0.80-1.00 [34].

We used logistic regression to estimate odds ratios (ORs) and 95% CIs for the associations of socio-demographic and health-related characteristics with agreement (Yes/No) for asthma status between the two sources. From administrative health databases, we used the asthma status based on the 30-year time interval. For self-reported asthma status, we considered self-report of ever having a physician diagnosis of asthma. Analyses were weighted by the inverse of the sampling probabilities to correct for the stratified sampling and variance was estimated by the Taylor series method in SAS PROC SURVEYLOGISTIC. We built the models by following the “purposeful selection of variables” approach described by Hosmer et al. [35]. We present estimates for the univariable models, the first multivariable model which includes all selected variables based on univariable associations (Wald statistic p-value < 0.25). For the most parsimonious model, we included variables with Wald statistic p-values < 0.25 in the full multivariable model. Some of the covariates had missing values, with the lowest observed for area of residence (corrected proportion, 0.7%) and highest for paternal education level (corrected proportion, 12%). Given the non-monotone missing pattern, we performed multiple imputations by the Markov Chain Monte Carlo method (20 imputed datasets) and applied logistic regression analyses on the imputed dataset.

We conducted four sets of sensitivity analyses. First, we assessed the effect of excluding some subjects from the interviewed subsample. When sampling was done for data collection, persons who had not met the asthma definition, but who had one physician claim for asthma between 1983 and 1994 were excluded. This may have resulted in artificially increasing the agreement for asthma status between administrative health data and self-report. If included, these subjects would have represented 5.9% of the eligible persons without asthma [36]. We re-analyzed the data, assuming two scenarios: (i) that they had been recruited in the same proportions as other subjects without asthma, and that all of them had reported having asthma whereas they were classified as not having asthma based on administrative health data (worst-case scenario), and (ii) that half of them had reported having asthma (intermediate scenario) [see Additional file 1]. Second, we assessed the effect of interruptions in provincial health insurance coverage which may have led to underestimating agreement since health services for asthma would have been sought outside of the province. Information on coverage interruptions were available from 1983 to 1994. We re-analyzed the data, excluding subjects with interruptions in provincial health insurance coverage, to ascertain whether a temporary lack of health coverage influenced the estimates. Third, we assessed asthma-related factors such as age at first and last service for asthma, time elapsed between first and last services, and asthma-related hospitalizations in relation to agreement. These analyses were conducted in a subset of 908 persons who had at least one medical service for asthma and who were classified as having asthma based on either administrative data or self-reported physician diagnosed asthma. Fourth, we conducted polytomous regression with the variables selected in the most parsimonious logistic regression model as determinants of agreement, to assess their specific associations with positive and negative agreement.

Statistical analyses were performed using SAS version 9.4 (SAS Institute Inc., Cary, NC, USA).

## Results

In the analytical sample (n = 1643), there were slightly more females (58%) than males, most participants were French speaking (94%), their parents were born in the province of Quebec (85%), and 60% lived exclusively in urban areas in 1987, 1991 and 2011 (Table 1). When correcting the distributions for the stratified sampling, the main differences were observed for parental history of asthma (subjects with parental history increased by 2% and missing values increased by 4%) and health services for allergic diseases (subjects without services increased by 13%).

**Table 1:** Selected characteristics of participants (N = 1643) and percentages corrected for the stratified sampling^a^

Characteristics	n	% in Survey sample	% Corrected for stratified sampling^a^
**Sex**			
Males	683	41.6	44.0
Females	960	58.4	56.0
**Language**			
French	1550	94.3	93.5
English	93	5.7	6.5
**Parental birthplace**			
In Quebec	1394	84.8	83.4
Outside Quebec	83	5.0	7.4
In and outside Quebec	93	5.7	4.9
Missing	73	4.4	4.3
**Area of residence 1987-2011** ^**b**^			
All urban	981	59.7	58.8
All rural	155	9.4	10.4
Urban and rural	501	30.5	30.1
Missing	6	0.4	0.7
**Family income 1991 and 2011** ^**c**^			
Quartile 1, on average	128	7.8	9.1
Quartile 2, on average	467	28.4	28.9
Quartile 3, on average	621	37.8	37.2
Quartile 4, on average	405	24.6	23.5
Missing	22	1.3	1.3
**Material deprivation index 1987-2011** ^**d**^			
1, most privileged	154	9.4	9.0
2	370	22.5	22.9
3	501	30.5	28.1
4	363	22.1	23.5
5, most deprived	91	5.5	5.5
Missing	164	10.0	11.1
**Social deprivation index 1987-2011** ^**d**^			
1, most privileged	135	8.2	8.3
2	472	28.7	29.7
3	455	27.7	26.0
4	348	21.2	21.0
5, most deprived	69	4.2	3.9
Missing	164	10.0	11.0
**Maternal education level**			
Elementary school	442	26.9	27.5
Secondary school	596	36.3	36.2
College	222	13.5	13.3
University	228	13.9	13.4
Missing	155	9.4	9.7
**Paternal education level**			
Elementary school	497	30.2	30.5
Secondary school	479	29.2	28.8
College	158	9.6	9.3
University	312	19.0	19.2
Missing	197	12.0	12.2
**Parental history of asthma**			
No	1390	84.6	78.5
Yes	220	13.4	15.5
Missing	33	2.0	6.0
**Number of health services for allergic diseases** ^**e**^			
0	711	43.3	55.9
≥ 1	932	56.7	44.1

^a^ Frequency distributions based on 1643 participants, corrected by applying weights corresponding to the inverse of the sampling probability from the *Quebec Birth Cohort on Immunity and Health* for the *Survey on Childhood Environment and the Development of Allergic Diseases* (stratified sampling among four strata, according to BCG vaccination status and asthma)

^b^ Determined using the second character of subjects’ residential postal codes (0: rural, ≠ 0: urban) in 1987, 1991 and 2011

^c^ Estimated by ‘median family income’ from the 1991 and 2011 Canadian census using the 1st three characters of subjects’ residential postal code. The summarized value consists in the average quartile of income over these two times, rounded up to the next integer

^d^ Estimated by the material and social deprivation indices based on the subjects’ residential postal codes in 1987, 1991 and 2011. The summarized value consists in the average index over these three times, rounded up to the next integer

^e^ Allergic diseases include allergic rhinitis, eczema, allergic urticaria, and other allergies non-specified; asthma is excluded

Compilation based on data from the ©Government of Quebec, *Institut de la statistique du Québec*, Survey on Childhood Environment and the Development of Allergic Diseases, 2012 and Quebec Birth Cohort on Immunity and Health, 2017. *Institut de la statistique du Québec* is not responsible for compilations or interpretation of results

Asthma prevalence ranged from 49% (algorithm within 1 year) to 55% (algorithm within 30 years) using administrative data, whereas the prevalence of ever having asthma was 49% and of ever having physician-diagnosed asthma was 46% according to self-report (Table 2). Once corrected for the stratified sampling, the prevalence ranged from 13 to 15% based on administrative data and from 16% (ever had physician-diagnosed asthma) to 19% (ever had asthma) based on self-report.

**Table 2:** Asthma prevalence according to data source for defining asthma

Source for asthma definition	Subjects with asthma n	Subjects with valid answers n	Prevalence^a^ %	Corrected prevalence^b^ %
Administrative databases (asthma-related health services)^c^				
≥ 2 PC within 1 year or ≥ 1 H	801	1643	48.8	12.8
≥ 2 PC within 2 years or ≥ 1 H	836	1643	50.9	13.7
≥ 2 PC within 3 years or ≥ 1 H	853	1643	51.9	13.9
≥ 2 PC within 5 years or ≥ 1 H	875	1643	53.3	14.5
≥ 2 PC within 30 years or ≥ 1 H	897	1643	54.6	15.0
Self-report				
Ever had asthma	807	1640	49.2	19.3
Ever had asthma diagnosed by a physician	762	1638	46.5	16.3

H, hospitalization; PC, physician claims

^a^ By design, the targeted prevalence of asthma among study participants was 50%

^b^ Corrected by applying weights corresponding to the inverse of the sampling probability from the *Quebec Birth Cohort on Immunity and Health* into the *Survey on Childhood Environment and the Development of Allergic Diseases* (stratified sampling among four strata, according to BCG vaccination status and asthma)

^c^ Lookback window from 1983 to 2012 (30 years)

Compilation based on data from the ©Government of Quebec, *Institut de la statistique du Québec*, Survey on Childhood Environment and the Development of Allergic Diseases, 2012 and Quebec Birth Cohort on Immunity and Health, 2017. *Institut de la statistique du Québec* is not responsible for compilations or interpretation of results

The proportions of overall and negative agreement between asthma defined from administrative data (5 algorithms) and based on two self-reported indicators were high, ranging from 88 to 91% and 93–95%, respectively (Table 3). The proportion of positive agreement was lower, ranging from 64 to 72%. Kappa values (0.57–0.67) indicated moderate to good agreement. The highest agreement was obtained with the algorithm considering ≥ 2 medical services over 30 years or ≥ 1 hospitalization, with a Kappa of 0.63 for the comparison with self-report of ever having asthma and of 0.67 with physician-diagnosed asthma. Agreement was slightly lower when definitions based on administrative data were compared with self-report of ever having asthma, than with self-reported asthma diagnosed by a physician.

**Table 3:** Agreement between asthma defined from administrative data and self-report among participants, with sampling weights applied

Asthma in administrative databases^a^		Self-report		% Agreement (95% CI)^b^	Kappa (95% CI)^b^	P pos (95% CI)^b^	P neg (95% CI)^b^
		**Ever had asthma (n = 1640)**					
		*Yes*	*No*				
≥ 2 PC within 1 year or ≥ 1 H	*Yes*	669	130	88.3 (86.8–89.9)	0.57 (0.52–0.63)	0.64 (0.59–0.69)	0.93 (0.92–0.94)
	*No*	138	703				
≥ 2 PC within 2 years or ≥ 1 H	*Yes*	698	136	89.0 (87.5–90.5)	0.60 (0.55–0.65)	0.67 (0.62–0.71)	0.93 (0.92–0.94)
	*No*	109	697				
≥ 2 PC within 3 years or ≥ 1 H	*Yes*	707	144	89.1 (87.6–90.6)	0.61 (0.56–0.66)	0.67 (0.63–0.72)	0.93 (0.92–0.94)
	*No*	100	689				
≥ 2 PC within 5 years or ≥ 1 H	*Yes*	723	150	89.0 (87.5–90.5)	0.61 (0.56–0.66)	0.67 (0.63–0.72)	0.93 (0.92–0.94)
	*No*	84	683				
≥ 2 PC within 30 years or ≥ 1 H	*Yes*	741	154	89.4 (87.9–90.9)	0.63 (0.58–0.68)	0.69 (0.65–0.74)	0.94 (0.93–0.95)
	*No*	66	679				
		**Asthma diagnosed by a physician (n = 1638)**					
		*Yes*	*No*				
≥ 2 PC within 1 year or ≥ 1 H	*Yes*	649	149	90.6 (89.2–92.0)	0.62 (0.57–0.68)	0.68 (0.63–0.72)	0.94 (0.94–0.95)
	*No*	113	727				
≥ 2 PC within 2 years or ≥ 1 H	*Yes*	676	156	90.9 (89.5–92.3)	0.64 (0.59–0.69)	0.70 (0.65–0.74)	0.95 (0.94–0.96)
	*No*	86	720				
≥ 2 PC within 3 years or ≥ 1 H	*Yes*	685	164	91.0 (89.6–92.4)	0.65 (0.60–0.70)	0.70 (0.66–0.75)	0.95 (0.94–0.96)
	*No*	77	712				
≥ 2 PC within 5 years or ≥ 1 H	*Yes*	700	171	90.9 (89.5–92.3)	0.65 (0.60–0.70)	0.70 (0.66–0.75)	0.95 (0.94–0.95)
	*No*	62	705				
≥ 2 PC within 30 years or ≥ 1 H	*Yes*	718	175	91.3 (90.0-92.7)	0.67 (0.62–0.72)	0.72 (0.68–0.77)	0.95 (0.94–0.96)
	*No*	44	701				

H, hospitalization; PC, physician consultation; P neg, proportion of negative agreement; P pos, proportion of positive agreement

^a^ Algorithms applied to asthma-related health services in a lookback window of 30 years (1983–2012)

^b^ All parameters are corrected for the stratified sampling and 95% CIs are estimated based on the actual number of survey respondents

Compilation based on data from the ©Government of Quebec, *Institut de la statistique du Québec*, Survey on Childhood Environment and the Development of Allergic Diseases, 2012 and Quebec Birth Cohort on Immunity and Health, 2017. *Institut de la statistique du Québec* is not responsible for compilations or interpretation of results

Analyses for identifying determinants of agreement were performed using the algorithm that considered a 30-year time interval to meet the definition in administrative health data (most permissive) and “physician-diagnosed asthma” from self-report (considered more valid), the two indicators leading to the strongest agreement. In univariable logistic regression analyses, language (p = 0.046), parental birthplace (p = 0.023), area of residence (p = 0.120), paternal education (p = 0.117), parental history of asthma (p = 0.079), and health services for allergic diseases other than asthma (p = 0.0001) met the criteria for inclusion (p ≤ 0.25) in the initial multivariable model (Table 4). From this model, only parental history of asthma and health services for other allergic diseases were kept in the final model (Table 4). Subjects who had any health services for allergic diseases other than asthma presented a lower likelihood of agreement compared with those who had no such health services (OR = 0.41, 95% CI: 0.25–0.65). Participants with parental history of asthma had a tendency toward lower likelihood of agreement (OR = 0.67, 95% CI: 0.37–1.23).

**Table 4:** Determinants of agreement between asthma defined from administrative data and self-report of ever having asthma diagnosed by a physician (n = 1638)^a^

Characteristics	No agreement n = 219 (column %)	Agreement n = 1419 (column %)	Univariable models^b^ OR (95% CI)	Multivariable initial model^b^ OR (95% CI)	Multivariable final model^b^ OR (95% CI)
**Sex**			*P = 0.289*		
Males	38.4	44.4	Ref		
Females	61.6	55.6	0.78 (0.50–1.23)		
**Language**			*P = 0.046*	*P = 0.266*	
French	87.8	94.0	Ref	Ref	
English	12.2	6.0	0.46 (0.21–0.98)	0.62 (0.26–1.45)	
**Parental birthplace**			*P = 0.023*	*P = 0.495*	
In Quebec	87.6	89.0	Ref	Ref	
Outside Quebec	5.7	5.2	0.49 (0.23–1.05)	0.72 (0.29–1.76)	
In and outside Quebec	6.7	5.8	0.60 (0.23–1.54)	0.62 (0.23–1.64)	
**Area of residence 1987-2011** ^**c**^			*P = 0.120*	*P = 0.417*	
All urban	62.6	58.9	Ref	Ref	
All rural	65.8	11.0	1.99 (0.81–4.94)	1.71 (0.65–4.51)	
Urban and rural	31.5	30.1	1.00 (0.62–1.62)	0.89 (0.53–1.50)	
**Family income 1991, 2011** ^**d**^			*P = 0.776*		
Quartile 1, on average	8.3	9.3	Ref		
Quartile 2, on average	29.4	29.3	0.89 (0.37–2.13)		
Quartile 3, on average	41.1	37.4	0.82 (0.35–1.91)		
Quartile 4, on average	21.1	23.9	1.03 (0.42–2.56)		
**Material deprivation index 1987-2011** ^**e**^			*P = 0.757*		
1, most privileged	9.6	10.2	Ref		
2	28.9	25.3	0.72 (0.26–1.92)		
3	27.0	32.0	0.89 (0.35–2.30)		
4	27.6	26.3	0.79 (0.30–2.08)		
5, most deprived	6.9	6.1	0.74 (0.21–2.62)		
**Social deprivation index 1987-2011** ^**e**^			*P = 0.801*		
1, most privileged	7.1	9.6	Ref		
2	32.2	33.4	0.84 (0.36–1.94)		
3	27.7	29.5	0.86 (0.37–1.98)		
4	28.1	23.2	0.73 (0.31–1.72)		
5, most deprived	4.8	4.3	0.70 (0.20–2.42)		
**Maternal education level**			*P = 0.436*		
Elementary school	27.0	30.8	Ref		
Secondary school	46.0	39.4	0.75 (0.41–1.40)		
College	12.0	15.0	1.02 (0.45–2.33)		
University	15.0	14.9	0.83 (0.38–1.81)		
**Paternal education level**			*P = 0.117*	*P = 0.353*	
Elementary school	40.8	34.2	Ref	Ref	
Secondary school	23.1	33.6	1.54 (0.86–2.76)	1.47 (0.77–2.81)	
College	8.9	10.7	1.36 (0.57–3.27)	1.32 (0.51–3.43)	
University	27.2	21.4	0.92 (0.50–1.72)	0.92 (0.46–1.83)	
**Parental history of asthma**			*P = 0.079*	*P = 0.222*	*P = 0.1982*
No	76.5	84.2	Ref	Ref	Ref
Yes	23.5	15.8	0.61 (0.35–1.06)	0.68 (0.37–1.26)	0.67 (0.37–1.23)
**Number of health services for allergic diseases** ^**f**^			*P = 0.0001*	*P = 0.0003*	*P = 0.0002*
0	32.4	45.0	Ref	Ref	Ref
≥ 1	67.6	55.0	0.40 (0.25–0.63)	0.42 (0.26–0.67)	0.41 (0.25–0.65)

^a^ Asthma from administrative health data was defined as having ≥ 2 asthma-related physician claims over 30 years or ≥ 1 asthma-related hospitalization, within a 30-year lookback window (1983–2012)

^b^ On imputed dataset and applying weights corresponding to the inverse of the sampling probability and correcting variance estimates with the Taylor series method

^c^ Determined using the second character of subjects’ residential postal codes (0: rural, ≠ 0: urban) in 1987, 1991 and 2011

^d^ Estimated by ‘median family income’ from the 1991 and 2011 Canadian census using the 1st three characters of subjects’ residential postal code. The summarized value consists in the average quartile of income over these two times

^e^ Estimated by the material and social deprivation indices based on the subjects’ residential postal codes in 1987, 1991 and 2011. The summarized value consists in the average index over these three times, rounded up to the next integer

^f^ Allergic diseases include allergic rhinitis, eczema, allergic urticaria, and other allergies non-specified; asthma is excluded

Compilation based on data from the ©Government of Quebec, *Institut de la statistique du Québec*, Survey on Childhood Environment and the Development of Allergic Diseases, 2012 and Quebec Birth Cohort on Immunity and Health, 2017. *Institut de la statistique du Québec* is not responsible for compilations or interpretation of results

### Sensitivity analyses

The first sensitivity analysis quantified the impact of having excluded persons who had not met the asthma definition, but who had one physician claim for asthma between 1983 and 1994. The resulting Kappa, representing the lowest Kappa that could have been obtained by including these subjects was 0.49 (95% CI: 0.44–0.54) and an intermediate scenario yielded a Kappa of 0.54 (95% CI: 0.49–0.59) [see Additional file 1]. The second sensitivity analysis addressed whether any discontinuous health insurance coverage could have influenced agreement between asthma definitions from administrative health data and self-report. Only 35 subjects (2%) had at least one period of ineligibility between 1983 and 1994. This information was not available beyond 1994. Results on agreement between administrative databases and self-report remained the same, and no differences were observed in results of logistic regression after excluding these subjects (data not shown). The third sensitivity analysis showed that, among subjects with asthma based either on administrative data or self-report, agreement was higher among those who were younger at their first health service for asthma: 88% at 8–11 years, 78% at 12–17 years, and 65% at ≥ 18 years. When considering age at last asthma-related health service, agreement increased from 55% at 8–11 years, to 60% at 12–17 years, and 84% at ≥ 18 years. Further, a longer duration between the first and last health service for asthma was related to a marked increase in agreement: 55% for 0–5 years, 83% for 6–10 years, 92% for 11–20 years, and 98% for ≥ 21 years. Agreement was also higher among those who had at least one asthma-related hospitalization as compared with those who had only physician claims (95% vs. 76% agreement, respectively). In the fourth sensitivity analysis, polytomous regression showed that negative agreement (agreement for not having asthma) was driving the observed associations [see Additional File 2].

## Discussion

We observed good agreement between administrative health data and self-reported ever asthma among adults who were close to their forties when they were interviewed. Of several sociodemographic and health-related characteristics considered, we found that not having sought health services for allergic diseases and, to a lesser extent, not having parental history of asthma were associated with better agreement between the two sources of information for asthma status.

Our study is the first to our knowledge to compare algorithms based on administrative health data gathered throughout most of the participants’ lives with self-report of ever having asthma among adults. Most previous studies have focused on administrative health data in the years just prior to the interview, which could explain the higher levels of agreement that we observed.

### Agreement

Nine studies were published by research groups in Canada [20–23, 25–27], the US [24], and Belgium [28] comparing administrative health data and self-report for ascertaining asthma status (see Additional file 3). Studies that used algorithms based on varying lookback windows (1–5 years) prior to the interview, found that analyses based on the longer lookback windows generated the highest Kappas [20–23]. When considering an administrative asthma definition that was met within a 1-year interval, Kappas ranged from 0.27 to 0.49 [20–24, 28]. The range of Kappas for definitions met within a 5-year interval was found to be higher, from 0.36 to 0.62 [20–23]. Three studies estimated Kappas by considering either a lookback period of 1 year [24, 28] or 2 years [26]. Among US veterans, a Kappa of 0.47 was estimated when considering physician claims and hospitalizations in the previous year [24]. In Belgium, a Kappa of 0.35 was observed between medication use and self-reported asthma over the prior year among persons aged ≥ 15 years [28]. In a study conducted in Quebec, Canada, a Kappa of 0.40 was estimated among persons aged < 65 years when considering health services in a 2-year lookback window prior to the time of self-report (asthma diagnosed by a physician or currently taking medication for asthma) [26].

The Kappas observed in our study were somewhat higher than those found in previous studies, ranging from 0.57 (over a 1-year time interval) to 0.63 (over a 30-year time interval), when considering self-report of ever having asthma. Agreement was higher with self-reported physician diagnosis of asthma, with Kappas ranging from 0.63 to 0.67 for a 1-year and a 30-year time interval, respectively. Like previous studies, there was a tendency toward a slightly increased agreement when the algorithm for administrative health data included a longer time interval. We used a longer lookback window in administrative health databases than any previous study (30 years, from age 8 to 38 years old) within which we considered either the full duration or time intervals of 1, 2, 3, or 5 years to meet the definition of asthma (≥ 2 physician claims or ≥ 1 hospitalization). In comparison, most previous studies have used algorithms applied to lookback windows of variable duration immediately before the interview [20–23, 26, 28]. Therefore, they were influenced by recent health care utilization, possibly reflecting asthma severity and control. The studies that are closest to ours in terms of lookback windows respectively found a Kappa of 0.55 (95% CI: 0.54–0.56) based on 10–15 years of administrative health data [25] and a Kappa of 0.47 (95% CI: 0.45–0.49) based on 12–14 years of administrative data [27]. Both used self-reported physician-diagnosed asthma and a definition from administrative health data that needed to be met within a 2-year interval, although the definitions differed slightly: ≥2 physician claims in 2 years or ≥ 1 hospitalization in the former, and ≥ 3 physician claims in 2 years or ≥ 1 hospitalization in the latter. In comparison, we observed stronger agreement (Kappa = 0.64 (95% CI: 0.59–0.69) than Muggah et al. [25] when applying their asthma definition to a 30-year lookback window of administrative health data. These results may suggest that a longer lookback window generates a more accurate determination of asthma status when applying definitions based on administrative health data. The longer lookback window may allow identifying childhood asthma that has resolved over time, for which participants would report having had asthma in the past. Incidentally, better concordance of diabetes status between administrative health data and medical records, has been reported for lookback windows of ≥ 10 years, compared to shorter ones [37].

When we sampled for participation in the Survey on Childhood Environment and the Development of Allergic Diseases, we excluded persons who had not met the asthma definition but who had one physician claim for asthma. In a sensitivity analysis, we observed that in the worse-case scenario, the resulting Kappa that could have been obtained by including them was slightly lower than the one that we originally estimated and closer to the values reported in previous studies. Thus, notwithstanding this methodological limitation, agreement would remain good in our study.

Neither administrative health data nor self-report is a gold standard for defining asthma status. However, both sources have previously been compared with medical records, considered as the gold standard. In Canada, administrative health data was found to be valid for identifying asthma when compared with medical charts, with a sensitivity of 84% (95% CI: 77–89) and a specificity of 76% (95% CI: 72–81) among individuals aged 19 years and over [9], and a sensitivity of 87% (95% CI: 80–94) and specificity of 94% (95% CI: 89–99) among 16–44 year-olds [14]. In practice, the quality of administrative health data can be affected by several factors including inadequate training or expertise of coding staff, systematic biases, problems related to transitions in coding systems or temporal changes, and problems related to data collection or coding strategies [38]. The validity of self-reported asthma, in comparison with medical records, was assessed in the US [15, 17–19] and UK [16]. Estimated kappa values ranged from moderate to good (0.57–0.78) [15–19]. The validity of self-report is likely affected by questionnaire wording, recall bias, and may differ according to socioeconomic status, education level, and health literacy [15]. Factors related to the validity of administrative health data and self-report for ascertainment of asthma status may explain some of the differences in agreement between these two indicators across different settings. Asthma ascertainment could alternatively be accomplished by applying natural language processing algorithms to electronic health records, if available [39–41].

### Determinants of agreement

Determinants of agreement were assessed in few studies. In Canada, male sex, age < 75 years (vs. older), absence of comorbid conditions (defined as allergies, emphysema, or chronic obstructive pulmonary disease), and higher income were associated with greater odds of agreement [23]. In Belgium, agreement was lower among persons aged 15–54 years and higher among those ≥ 75 years age, compared with 55-74-year-olds. Agreement was also related to higher education levels, better perceived health, and absence of comorbidities (undefined) [28]. Some studies assessed determinants of agreement between self-report and medical charts. Among inner city seniors in New York, better agreement was observed among those with higher income and better general health [17]. Female sex, younger age, and higher education were also shown to be related to stronger agreement in a population of American Indians and Alaska Natives [19]. Interestingly, upon assessing agreement between parental self-report and children’s utilization of health services for asthma in the US, co-occurring allergies (seasonal, respiratory, food allergies, and eczema) were found to be related to lower agreement [42]. This is in line with our findings, which further suggest that use of health services for allergic diseases is related to lower agreement as compared with no use. Among all the potential predictors that we assessed, this was the strongest (negative) predictor of agreement. Our results from polytomous regression shed some light toward the interpretation of this determinant. Having had health services for allergic diseases is not associated with positive agreement (having asthma), but rather with lower agreement for a “non-asthma” status. In other words, the absence of health services for asthma is related to a higher likelihood of agreement for absence of asthma. One noteworthy aspect is the paucity of potential determinants that we identified among the large number of sociodemographic and individual characteristics considered, notably the absence of association with sex and with most of the sociodemographic variables considered. Sensitivity analyses allowed us to show that positive agreement was related to characteristics of asthma. Agreement was higher among participants with a longer duration of asthma (as compared with shorter) and those who had at least one asthma-related hospitalization (vs. only physician claims). This suggests that sustained asthma over time, as well as more severe and/or less controlled asthma were more accurately recorded in administrative data and reported.

### Study limitations

Some limitations should be acknowledged. Discordance between administrative data and self-reported asthma may be due to participants confusing asthma with other respiratory conditions, such as chronic bronchitis, emphysema, COPD. In addition, alternative billing codes could have been used by physicians in situations of uncertainty about the diagnosis. Unfortunately, the alternative billing codes were not available in the datasets, which hampered our ability to conduct sensitivity analyses. However, such misclassification is unlikely given the relatively young age of the population studied and the low prevalence of these conditions in young adults.

No administrative health data was available before 1983, when participants were 8–9 years old. Thus, for cases of wheezing in infancy that completely resolved and did not lead them to seek further medical attention for asthma, subjects may have reported ever having asthma but would not have been identified in administrative data.

The exclusion of subjects who had only one physician claim for asthma between 1983 and 1994 is a potential limitation of this study. Our sensitivity analysis showed that agreement may have been slightly overestimated, but that it would have been good, nonetheless, if we had included these subjects.

We could not assess education or income as potential determinants of agreement since individual data on education and income were not collected. We addressed this lack of information by using contextual variables based on postal code of residence and census data (income, material and social deprivation indices), as well as parents’ level of education. Age could not be investigated either, because all participants were born in the same calendar year.

## Conclusion

Our findings suggest a good agreement between asthma status defined from administrative databases and self-report of having ever had this condition. A longer lookback window may result in more accurate determination of ever asthma status when applying definitions based on administrative health data. Not having sought health services for allergic diseases, and to a lesser extent, not having parental history of asthma were related to better agreement between administrative databases and self-reported physician-diagnosed asthma. This research supports the use of both administrative health data and self-report for ascertaining asthma in epidemiological studies.

### Electronic supplementary material

Below is the link to the electronic supplementary material.

Supplementary Material 1

Supplementary Material 2

Supplementary Material 3

## Data Availability

The data that support the findings of this study are not publicly available. Access, in secured data centres, requires permission of *Institut de la statistique du Québec* and *Commission d’Accès à l’Information* of Quebec. Requests should be directed to the corresponding author who will contact the relevant authorities at *Institut de la statistique du Québec*.
