# Supplementary material for: Ascertaining asthma status in epidemiologic studies: a comparison between administrative health data and self-report
Source: BMC Med Res Methodol. 2023 Sep 7;23:201. doi: 10.1186/s12874-023-02011-6 (PMC10486089; doi:10.1186/s12874-023-02011-6)
Supplement: Supplementary file 1 — Supplementary Material 1 [file 12874_2023_2011_MOESM1_ESM.docx]

**Additional file 1**

Sensitivity analyses aimed at assessing the impact of having excluded persons with uncertain asthma status from the subset of subjects interviewed.

There were 4,457 persons excluded from pool of eligible subjects for data collection because they did not meet the definition of asthma, but had one physician claim for asthma [1].

BCG^+^ / Asthma^–^ 33,040

BCG^–^ / Asthma^–^ 37,713

Asthma^–^ excluded 4,457

Total 75,210

This represents a proportion of 5.9% (4,457/75,210) of all subjects without asthma (according to administrative data until 1994).

The data were re-analyzed, assuming that 5.9% of persons without asthma according to administrative databases and who participated in the telephone interviews, were actually persons with one physician claim for asthma and would have reported having asthma. As shown in the Table below, this amounts to transferring 48 persons ((109+697)*0.059) from the d cell to the c cell in the unweighted analysis, and to transferring 3895 persons ((6359+59,661)*0.059) from the d cell to the c cell in the weighted analysis (with a theoretical sample size of 76,502). The proportion of agreement and Kappa were estimated in the weighted sample, but the 95% confidence intervals are based on the actual number of participants. This is a worse-case scenario, in which all the persons who have been excluded would have been discordant for their asthma status. In this worse-case scenario, the overall proportion of agreement decreases from 89% to 84% and Kappa decreases from 0.60 to 0.49.

If, in an intermediate scenario, 3% of all subjects without asthma were transferred from the d to c cell (instead of 5.9%), the proportion of agreement would be 86% and the Kappa 0.54.

| **Asthma in administrative databases^a^** |  | **Self-report, Ever had asthma** | | | | **% Agreement**  **(95% CI)** | **Kappa**  **(95% CI)** |
| --- | --- | --- | --- | --- | --- | --- | --- |
|  |  | **Unweighted**  **(n=1640)** | | **Weighted**  **(theoretical n=76,502)** | |  |  |
|  |  | *Yes* | *No* | *Yes* | *No* |  |  |
| Original analysis | *Yes* | a 698 | b 136 | a 8422 | b 2060 | 89.0 (87.5-90.5) | 0.60 (0.55-0.65) |
|  | *No* | c 109 | d 697 | c 6359 | d 59,661 |  |  |
|  |  |  |  |  |  |  |  |
| Assuming an additional 5.9% of non-asthma would self-report having had asthma instead of no asthma | *Yes* | 698 | 136 | 8422 | 2060 |  |  |
|  | *No* | 109+48 | 697-48 | 6359+3895 | 59,661-3895 |  |  |
|  |  |  |  |  |  |  |  |
|  | *Yes* | 698 | 136 | 8422 | 2060 | 83.9 (82.1-85.7) | 0.49 (0.44-0.54) |
|  | *No* | 157 | 649 | 10,254 | 55,766 |  |  |
|  |  |  |  |  |  |  |  |
| Assuming an additional 3% of non-asthma would self-report having had asthma instead of no asthma | *Yes* | 698 | 136 | 8422 | 2060 |  |  |
|  | *No* | 109+24 | 697-24 | 6359+1981 | 59,661-1981 |  |  |
|  |  |  |  |  |  |  |  |
|  | *Yes* | 698 | 136 | 8422 | 2060 | 86.4 (84.7-88.1) | 0.54 (0.49-0.59) |
|  | *No* | 133 | 673 | 8340 | 57,680 |  |  |

^a^ Analyses based on the asthma definition [≥ 2 physician claims in 2 years or ≥ 1 hospitalization] used in the largest number of previous studies.

Compilation based on data from the ©Government of Quebec, Institut de la statistique du Québec, Survey on Childhood Environment and the Development of Allergic Diseases, 2012. Institut de la statistique du Québec is not responsible for compilations or interpretation of results.

1. El-Zein M, Conus F, Benedetti A, Menzies D, Parent ME, Rousseau MC: **Association between Bacillus Calmette-Guérin (BCG) vaccination and childhood asthma in the Québec Birth Cohort on Immunity and Health**. *Am J Epidemiol* 2017, **186**(3):344-355.
