# Supplementary material for: Ascertaining asthma status in epidemiologic studies: a comparison between administrative health data and self-report
Source: BMC Med Res Methodol. 2023 Sep 7;23:201. doi: 10.1186/s12874-023-02011-6 (PMC10486089; doi:10.1186/s12874-023-02011-6)
Supplement: Supplementary file 2 — Supplementary Material 2 [file 12874_2023_2011_MOESM2_ESM.docx]

**Additional file 2**

**Sensitivity analysis – Polytomous regression models for determinants of positive and negative agreement between asthma defined from administrative data and self-report of ever having asthma diagnosed by a physician (n=1638)^a^**

| **Characteristics** | **Positive agreement^b^**  **OR (95% CI)** | **Negative agreement^b^**  **OR (95% CI)** |
| --- | --- | --- |
| **Parental history of asthma** | *P=0.151* | *P=0.056* |
| No | Ref | Ref |
| Yes | 1.60 (0.84-3.02) | 0.54 (0.29-1.02) |
|  |  |  |
| **Number of health services for allergic diseases^c^** | *P=0.789* | *P<0.0001* |
| 0 | Ref | Ref |
| ≥1 | 1.08 (0.62-1.87) | 0.35 (0.22-0.56) |

^a^ Asthma from administrative health data was defined as having ≥2 asthma-related physician claims over 30 years or ≥1 asthma-related hospitalization, within a 30-year lookback window (1983-2012). There were 219 subjects without agreement, 718 with positive agreement (asthma) and 701 with negative agreement (no asthma).

^b^ On imputed dataset and applying weights corresponding to the inverse of the sampling probability and correcting variance estimates with the Taylor series method.

^c^ Allergic diseases include allergic rhinitis, eczema, allergic urticaria, and other allergies non-specified; asthma is excluded.

Interpretation: A tendency toward an increased likelihood of positive agreement was observed among those with parental history of asthma, whereas there was no association between the number of health services for allergic diseases other than asthma and positive agreement. Having a parental history of asthma and having had health services for allergic diseases (excluding asthma) were associated with a lower likelihood of negative agreement. In other words, when considering the inverse of these odds ratios for ease of interpretation, not having parental history of asthma was associated with a twofold likelihood of negative agreement (vs. having parental history) and not having received health services for allergic diseases other than asthma was associated with a threefold likelihood of negative agreement (vs. having received some health services for allergic diseases).

Compilation based on data from the ©Government of Quebec*, Institut de la statistique du Québec*, Survey on Childhood Environment and the Development of Allergic Diseases, 2012 and Quebec Birth Cohort on Immunity and Health, 2017. *Institut de la statistique du Québec* is not responsible for compilations or interpretation of results.
