# Supplementary material for: Ascertaining asthma status in epidemiologic studies: a comparison between administrative health data and self-report
Source: BMC Med Res Methodol. 2023 Sep 7;23:201. doi: 10.1186/s12874-023-02011-6 (PMC10486089; doi:10.1186/s12874-023-02011-6)
Supplement: Supplementary file 3 — Supplementary Material 3 [file 12874_2023_2011_MOESM3_ESM.docx]

**Additional file 3**

**Summary of studies on agreement between self-report and administrative health data for ascertaining asthma, according to selected asthma definitions from administrative data**

| **Study, Country** | **Age** | **Administrative data definition**  **Lookback window** | **Within 1 year**  **Kappa (95% CI)** | **Within 2 years**  **Kappa (95% CI)** | **Within 3 years**  **Kappa (95% CI)** | **Within 5 years**  **Kappa (95% CI)** |
| --- | --- | --- | --- | --- | --- | --- |
| Huzel 2002[4] Manitoba, Canada | 20-44 years | ≥1 PC  Lookback window: 1, 2, or 5 years prior to interview | 0.49 (0.42-0.56) | 0.59 (0.53-0.66) | – | 0.62 (0.56-0.68) |
| Question: “Have you had an attack of asthma in the last 12 months?” or “Are you currently taking any medication (including inhalers, aerosols or tablets) for asthma?” | | | | | | |
| Lix 2006[5]  Manitoba, Canada | 19-49 years | ≥2 PC or ≥1 H  Lookback window: 1, 2, 3, or 5 years prior to interview | 0.27 (0.20-0.34) | 0.26 (0.18-0.33) | 0.24 (0.17-0.30) | 0.36 (0.29-0.42) |
| Question: “Now I’d like to ask about certain chronic health conditions which you may have. We are interested in ‘long-term conditions’ that have lasted or are expected to last six months or more and that have been diagnosed by a health professional. Do you have asthma?” | | | | | | |
| Lix 2008[6]  Manitoba, Canada | ≥19 years | ≥2 PC or ≥1 H  Lookback window: 1, 2, 3, or 5 years prior to interview | 0.27 (0.27-0.27) | 0.35 (0.35-0.35) | 0.43 (0.43-0.43) | 0.48 (0.48-0.49) |
| Question: “Now I’d like to ask about certain chronic health conditions which you may have. We are interested in ‘long-term conditions’ that have lasted or are expected to last six months or more and that have been diagnosed by a health professional. Do you have asthma?” | | | | | | |
| Lix 2008[7]  Manitoba, Canada | ≥12 years | ≥1 PC or ≥1 H  Lookback window: 1, 2, 3, or 5 years prior to interview | 0.40 (0.36-0.45) | 0.50 (0.46-0.54) | 0.53 (0.50-0.57) | 0.55 (0.51-0.59) |
| Question: “Now I’d like to ask about certain chronic health conditions which you may have. We are interested in ‘long-term conditions’ that have lasted or are expected to last six months or more and that have been diagnosed by a health professional. Do you have asthma?” | | | | | | |
| Singh 2009[8]  US | Adults (veterans) | ≥1 PC or ≥1 H  Lookback window: year before and year after survey | 0.47 (0.47-0.48) | – | – | – |
| Question: “Has your doctor ever told you that you have asthma?” | | | | | | |
| Muggah 2013[9] Ontario, Canada | ≥20 years | ≥2 PC in 2 years or ≥1 H  Lookback window: 1991 to survey date (2001, 2003 or 2005), 10-15 years | – | 0.55 (0.54-0.56) | – | – |
| Question: …“long-term conditions that have lasted or are expected to last six months or more and that have been diagnosed by a health professional. Do you have asthma?” | | | | | | |
| Fortin 2017[10]  Quebec, Canada | <65 years | ≥1 PC or ≥1 H  Lookback window: 2-year period prior to interview | – | 0.40 (0.29-0.50) | – | – |
| Question: “Have you been told by a health professional that you have asthma OR do you take medication to control asthma?” | | | | | | |
| Payette 2020[11]  Quebec, Canada | 40-69 years | ≥3 PC in 2 years or less or ≥1 H  Lookback window: 1998 to one year after consent (2010-2012), 12-14 years | – | 0.47 (0.45-0.49) | – | – |
| Question: “Has a doctor ever told you that you had asthma?” | | | | | | |
| Berete 2020[12] Belgium | ≥15 years | Medication use, daily defined dose ≥90  Lookback window: 12 months prior to interview | 0.35 (0.30–0.41) | – | – | – |
| Question: “Have you suffered during the last 12 months from asthma?” | | | | | | |

H: hospitalization; PC: physician consultation

**REFERENCES**

4. Huzel L, Roos LL, Anthonisen NR, Manfreda J: **Diagnosing asthma: the fit between survey and administrative database**. *Can Respir J* 2002, **9**(6):407-412.

5. Lix L, Yogendran M, Burchill C, Metge C, McKeen N, Moore D, Bond R: **Defining and validating chronic diseases: an administrative data approach**. In*.*: Manitoba Centre for Health Policy, University of Manitoba; 2006.

6. Lix L, Yogendran M, Mann J: **Defining and validating chronic diseases: an administrative data approach. An udpate with ICD-10-CA**. In*.*: Manitoba Centre for Health Policy, University of Manitoba; 2008: 79.

7. Lix LM, Yogendran MS, Shaw SY, Burchill C, Metge C, Bond R: **Population-based data sources for chronic disease surveillance**. *Chronic Dis Can* 2008, **29**(1):31-38.

8. Singh JA: **Accuracy of Veterans Affairs databases for diagnoses of chronic diseases**. *Prev Chronic Dis* 2009, **6**(4):A126.

9. Muggah E, Graves E, Bennett C, Manuel DG: **Ascertainment of chronic diseases using population health data: a comparison of health administrative data and patient self-report**. *BMC Public Health* 2013, **13**:16.

10. Fortin M, Haggerty J, Sanche S, Almirall J: **Self-reported versus health administrative data: implications for assessing chronic illness burden in populations. A cross-sectional study**. *CMAJ open* 2017, **5**(3):E729-E733.

11. Payette Y, de Moura CS, Boileau C, Bernatsky S, Noisel N: **Is there an agreement between self-reported medical diagnosis in the CARTaGENE cohort and the Quebec administrative health databases?** *International journal of population data science* 2020, **5**(1):1155.

12. Berete F, Demarest S, Charafeddine R, Bruyere O, Van der Heyden J: **Comparing health insurance data and health interview survey data for ascertaining chronic disease prevalence in Belgium.** *Arch Public Health* 2020, 78(1):120.
